# Predictors of Urosepsis Post Percutaneous Nephrolithotomy at King Abdulaziz Medical City, Riyadh

**DOI:** 10.7759/cureus.39542

**Published:** 2023-05-26

**Authors:** Fahad S Alhamad, Abdulaziz Alathel, Ziad A Aljaafri, Khalid H Alhadlaq, Abdullah Alghamdi, Yazeed S AlHoshan, Omar Alfraidi

**Affiliations:** 1 College of Medicine, King Saud Bin Abdulaziz University for Health Sciences, Riyadh, SAU; 2 Urology, King Saud Bin Abdulaziz University for Health Sciences, Riyadh, SAU; 3 Urology, Ministry of National Guard-Health Affairs, King Abdulaziz Medical City, Riyadh, SAU; 4 Collage of Medicine, King Saud Bin Abdulaziz University for Health Sciences, Riyadh, SAU

**Keywords:** nephrolithotomy, percutaneous, urosepsis, risk factors, patients

## Abstract

Introduction: The procedure of percutaneous nephrolithotomy (PCNL) is considered a minimally invasive method for removing stones from the kidneys or ureters. PCNL can cause a wide range of complications, such as urosepsis, a rare but serious complication.

Methods: A retrospective cohort study of patients who underwent PCNL from the period 2016 to 2022 was conducted at King Abdulaziz Medical City. Data were collected by chart review using the BestCARE system. SPSS version 23 (IBM Corporation, Armonk, NY, USA) was used. Qualitative variables were expressed as percentages and frequencies. The chi-square test was used to compare the qualitative variables. The K-S test was used to check the normality of the data. Quantitative variables were compared between groups using the independent sample t-test and the nonparametric Mann-Whitney test. Fisher's exact test was used to compare categorical variables.

Results: A total of 155 patients were included in this study. The mean age of the participants overall was found to be 49. About 108 (69.7%) of the participants were male. Regarding risk factors for urosepsis, diabetes mellitus was found in 54 (34.8%) of the participants. The incidence of urosepsis following PCNL was found to be 3 (1.9%) of the patients. The most frequently reported indication was found to be unilateral renal stones. The most frequently reported type of stone in the analysis was found to be calcium oxalate in nearly two-thirds 98 (63.2%) of the patients.

Conclusion: The incidence of urosepsis among the patients who underwent PCNL was less than 2%. Diabetes mellitus, followed by hypertension, were the most prevalent co-morbidities among the participants. Cefuroxime was the antibiotic of choice when treating patients and following urosepsis.

## Introduction

Kidney stones are a common urological condition that affects approximately 12% of the world's population. Percutaneous nephrolithotomy (PCNL) is considered a minimally invasive procedure used to remove large kidney or ureteric stones [1]. PCNL can result in a variety of complications, including bleeding, injury to the surrounding structure, infection, positioning-related injuries, thromboembolic diseases, and even death [2]. Urosepsis is a rare but serious complication after PCNL. Sometimes, urosepsis can cause septic shock and be lethal if left untreated or not treated properly [3-4]. The most common bacteria associated with urosepsis are Escherichia coli, Proteus, Klebsiella, and Pseudomonas spp., among others that are less frequent [5].

In practice, the first stage of urosepsis often lacks clinical symptoms, which makes it difficult to identify the occurrence of urosepsis at an early stage [6]. Many studies have shown that there are many risk factors for urosepsis after PCNL, including preoperative, intraoperative, and postoperative factors. Positive urine culture, stone size, stone complexity, and residual stones were the main risk factors for urosepsis [7-8].

Due to a lack of studies evaluating risk factors associated with urosepsis after PCNL, we decided to evaluate risk factors that are associated with urosepsis in patients who underwent PCNL at King Abdulaziz Medical City, Riyadh.

## Materials and methods

A retrospective study was conducted at King Abdulaziz Medical City (KAMC), a tertiary hospital in Riyadh. The study included all patients who underwent PCNL between January 2016 and December 2022. Both genders were included. Exclusion criteria were applied for patients who underwent the procedure outside the institution and patients with missing data. Non-probability consecutive sampling included all patients who met the inclusion criteria.

The data were collected through the BESTCare system at KAMC. The main categories of the sheet used included the following: demographic data, surgery data, comorbidities, hospital course that includes ICU admission, antibiotic course, stone analysis, and post-discharge data that includes antibiotic and emergency room visits, time of JJ stent removal, residual stone, and subsequent intervention.

Data were collected through Microsoft Excel (Microsoft, Redmond, WA, USA) and transferred to Statistical Package for Social Sciences (SPSS) Statistics v.23 (IBM Corp., Armonk, NY, USA) for statistical analysis. The data were checked for missing information, and new variables were recorded and computed based on the extracted data. Once the distribution of the variables was determined, appropriate correlation analyses were chosen. All assumptions were satisfied for each analysis. Moreover, qualitative variables were expressed as percentages and frequencies. A chi-square test was used to compare the qualitative variables. The K-S test was used to check the normality of the data. Quantitative variables were compared between groups using the independent sample t-test and the nonparametric Mann-Whitney test. Fisher's exact test was used to compare categorical variables. A P-value of 0.05 was considered significant.

Consent was not required because this was a retrospective cohort study and all data were kept safe. No identification data were asked, such as medical record numbers (MRN), names, or IDs. Subjects' privacy and confidentiality were assured; no identifiers were collected, and all data were kept in a secure place within the National Guard Health Affairs (NGHA) premises, both in hard and soft copies. Access to research data was kept only between the study group members.

## Results

A total of 155 patients were included in this study. The mean age of the patients overall was found to be 49.9 ± standard deviation (SD) of 18.5 years (range of 6-88 years). The mean age of the patients with urosepsis was found to be 40.7 ± 31 years old. The mean age of the patients with no urosepsis was found to be 50.1 ± 18.3. The mean BMI overall was found to be 29.3 ± 7.3, the mean BMI for patients with urosepsis was found to be 18.9 ± 7.8, the mean BMI for patients without urosepsis was found to be 29.5 ± 7.1, and BMI was found to be significant with urosepsis (p-value = 0.012), as patients with a higher BMI were found to develop urosepsis less frequently than those with a normal or low BMI. About 108 (69.7%) of the participants were males and 47 (30.3%) were females; 3 (100%) of the patients who developed urosepsis were males; the rest of the males, 105 (69.1%), had no urosepsis; females, 105 (30.9%), also had no urosepsis; no significant association was found between gender and urosepsis (p-value = 0.554).

Regarding risk factors for urosepsis, diabetes mellitus was found in 54 (34.8%) of the patients. One (33%) of the patients with urosepsis was found to be diabetic; there was no significant difference between diabetes mellitus and urosepsis (p-value = 1.000). Fifty-seven (36.8%) were hypertensive, and only one (33%) of those who were found to have urosepsis were hypertensive; no significant association was found between hypertension and urosepsis (p-value = 1.000). Fourteen (9%) were found to have chronic kidney disease; nine (5.8%) had cardiovascular diseases; and six (3.9%) had hematological disorders. Five (3.2%) had steroid use; there was no difference between the patients using steroids and those with urosepsis (p-value = 1.000). About 64 (41.3%) had other co-morbidities, and 2 (66%) of those with urosepsis were found to have other co-morbidities. There is no significant association between other co-morbidities and urosepsis (p-value = 0.569). Table 1 shows the demographics and risk factors for all the patients developing urosepsis following PCNL.

**Table 1:** Demographic data, patients’ risk factors, and its association with developing urosepsis following PCNL (n=155) T: p-value calculated using independent samples t-test, other p-values calculated using Fisher's exact test.

Variable	Overall	Urosepsis		P-value
		Yes	No	
Age: mean ± SD (range)	49.9 ± 18.5 years (6 – 88)	40.7 ± 31.0	50.1 ± 18.3	0.386^T^
BMI: mean ± SD	29.3 ± 7.3	18.9 ± 7.8	29.5 ± 7.1	0.012^T^
Gender: n (%)				
Male	108 (69.7)	3 (100)	105 (69.1)	0.554
Female	47 (30.3)	0 (0)	47 (30.9)	
Patient risks factors: n (%)				
Diabetes mellitus	54 (34.8)	1 (33.3)	53 (34.9)	1.000
Hypertension	57 (36.8)	1 (33.3)	56 (36.8)	1.000
Hypothyroidism	3 (1.9)	0 (0)	3 (2)	1.000
Hematological disorder	6 (3.9)	0 (0)	6 (3.9)	1.000
Cardiovascular disease	9 (5.8)	0 (0)	9 (5.9)	1.000
Chronic kidney disease	14 (9)	0 (0)	14 (9.2)	1.000
Steroid use	5 (3.2)	0 (0)	5 (3.3)	1.000
Other comorbidities	64 (41.3)	2 (66.7)	62 (40.8)	0.569

**Figure 1:**
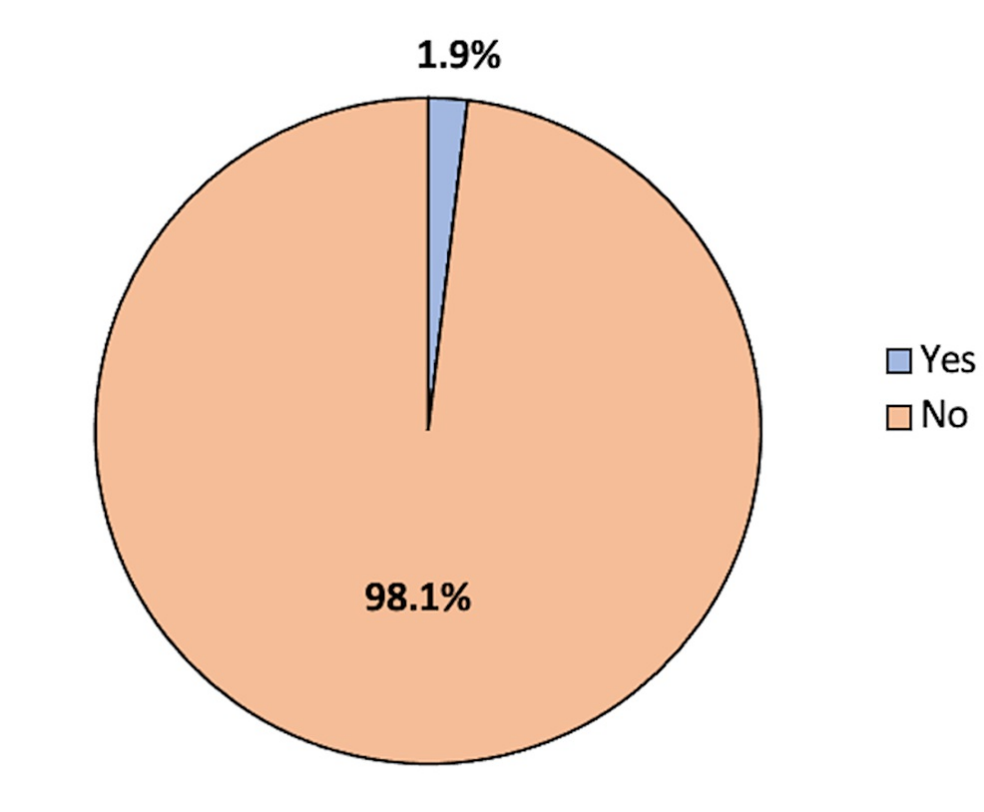
Incidence of urosepsis following percutaneous nephrolithotomy

The incidence of urosepsis following PCNL was found to be 3 (1.9%) of the participants, and the rest (98.1%) had no urosepsis (Figure 1).

Concerning the diagnosis and indication of PCNL, the most frequently reported indication was found to be unilateral renal stones in 114 (73.5%) patients, and all 3 (100%) of those with urosepsis were found to have unilateral renal stones; staghorn stones were found in 24 (15.5%) patients; 10 (6.5%) patients had bilateral renal stones; and 7 (4.5%) had ureteric stones. No statistically significant association was found between indications of PCNL and urosepsis (p-value = 0.746).

The mean age at the time of surgery was found to be 46.4 ± 18.1 years old; the mean age at the time of surgery for patients with urosepsis was found to be 37.3 ± 30.3 years old; and the mean age at the time of surgery for patients with no urosepsis was found to be 46.6 ± 17.9 years old (p-value = 0.384). Complications of PCNL were reported in two (1.3%) patients; complications were found among those with no urosepsis. Previous PCNL was reported in 31 (20%) patients. Five (3.2%) patients were admitted to the ICU; three (100%) of those with urosepsis were admitted to the ICU. ICU admission and urosepsis were found to be significantly associated (p-value < 0.001), with patients with urosepsis being more likely to be admitted to the ICU compared to others. The mean length of stay (in days) was found to be 7.7 ± 11.5 in general; the mean length of stay for those with urosepsis was found to be 52.3 ± 34.9 days; and the length of stay for those with no urosepsis was found to be 6.8 ± 8.8 days. Sixty-four (41.3%) patients had an antibiotic treatment course; all three (100%) patients with urosepsis were on an antibiotic treatment course; 61 (40.1%) patients with no urosepsis were on an antibiotic treatment course; and no significant association was found between the antibiotic treatment course and urosepsis (p-value = 0.068).

The most frequently reported type of stone in the analysis was found to be calcium oxalate in nearly two-thirds, or 98 (63.2%), of the patients; urate stones were found in 24 (15.5%) patients, cystine, calcium oxalate, and carbonate were found in 9 (5.8%) patients each; and struvite stones were reported in 6 (3.9%) patients. Less than one-third, 45 (29%), of the patients had no antibiotics upon discharge, and 1 (33.3%) of those with urosepsis were discharged without antibiotics. About 45 (29%) received cefuroxime, and 1 (33.3%) of those with urosepsis also received cefuroxime; 44 (15.8%) of the patients with no urosepsis have received cefuroxime; 42 (27.1%) of the overall patients received ciprofloxacin; none of those with urosepsis received ciprofloxacin; bactrim was given to 13 (8.4%) patients; and also none of the participants with urosepsis received bactrim; and norfloxacin was used in the treatment of 6 (3.9%) of the patients, and none of the patients with urosepsis received norfloxacin. Nitrofurantoin was used by 4 (2.6%) of the patients, and 1 (33%) patient with urosepsis received nitrofurantoin in the treatment course. The mean duration of antibiotic treatment among overall patients was found to be 8.4 ± 10.1 days; the mean antibiotic treatment among patients with urosepsis was found to be 16.5 ± 16.3 days; and among those with no urosepsis, it was found to be 8.3-10 days.

About 49 (31.6%) of the overall patients had post-operative ER visits, and 1 (33%) of those with urosepsis had a post-operative ER visit. Time of removal of JJ stent was found to be more than two weeks in 88 (56.8%) patients, and 1 (33%) of the patients with urosepsis had JJ stent inserted for more than two weeks; about 16 (10.3%) of the patients had JJ stent inserted for more than one week but less than two weeks; also, 1 (33%) of the patients with urosepsis had JJ stent inserted for more than one week but less than two weeks; 11 (7.1%) had a time of removal of less than one week; and time of removal was not found to be significantly associated with urosepsis (p-value = 0.748).

Residual stone was found in 70 (45.2%) patients, and all 3 (100%) of the patients with urosepsis had residual stone; 53 (34.2%) patients had no residual stone, and 32 (20.6%) patients had no CT done. More than one-third, 59 (38.1%), of the patients had a subsequent intervention, and 2 (66%) of the patients with urosepsis had a subsequent intervention. Subsequent intervention and urosepsis were not found to be significantly associated (p-value = 558). In Table 2, surgery, hospital course, and post-discharge status were described.

**Table 2:** Information of percutaneous nephrolithotomy surgery, hospital course, and data upon discharge and its association with urosepsis M: p-values calculated using Mann-Whitney test, T: independent samples t-test, other p-values calculated using Fisher's exact test; PCNL: percutaneous nephrolithotomy; ICU: intensive care unit; ER: emergency room.

Variable	Overall n (%)	Urosepsis		P-value
		Yes	No	
Indications/diagnosis				
Bilateral renal stone	10 (6.5)	0 (0)	10 (6.6)	0.746
Staghorn stone	24 (15.5)	0 (0)	24 (15.8)	
Unilateral renal stone	114 (73.5)	3 (100)	111 (73)	
Ureter stone	7 (4.5)	0 (0)	7 (4.6)	
Age at time of surgery (years): mean ± SD	46.4 ± 18.1	37.3 30.3	46.6 17.9	0.384^T^
Complications	2 (1.3)	0 (0)	2 (1.3)	1.000
Previous PCNL	31 (20)	0 (0)	31 (20.4)	0.612
ICU admission	5 (3.2)	3 (100)	2 (1.3)	< 0.001
Length of stay (days): mean ± SD	7.7 ± 11.5	52.3 34.9	6.8 8.8	0.004^M^
Antibiotic course	64 (41.3)	3 9100)	61 (40.1)	0.068
Stone analysis				
Calcium oxalate	98 (63.2)	2 (66.7)	96 (63.2)	0.089
Cystine	9 (5.8)	0 (0)	9 (5.9)	
Urate	24 (15.5)	0 (0)	24 (15.8)	
Calcium oxalate and urate	6 (3.9)	0 (0)	6 (3.9)	
Calcium oxalate and cystine	3 (1.9)	1 (33.3)	2 (1.3)	
Calcium oxalate and carbonate	9 (5.8)	0 (0)	9 (5.9)	
Struvite	6 (3.9)	0 (0)	6 (3.9)	
Antibiotic upon discharge				
No	45 (29)	1 (33.3)	44 (28.9)	0.098
Bactrim	13 (8.4)	0 (0)	13 (8.6)	
Cefuroxime	45 (29)	1 (33.3)	44 (28.9)	
Norfloxacin	6 (3.9)	0 (0)	6 (3.9)	
Nitrofurantoin	4 (2.6)	1 (33.3)	3 (2)	
Ciprofloxacin	42 (27.1)	0 (0)	42 (27.6)	
Duration of antibiotics (days): mean ± SD	8.4 ± 10.1	16.5 16.3	8.3 10.0	0.524^M^
ER visit post-op	49 (31.6)	1 (33.3)	48 (31.6)	1.000
Time of removal of JJ stent				
N/A	40 (25.8)	1 (33.3)	39 (25.7)	0.748
<1 week	11 (7.1)	0 (0)	11 (7.2)	
>1 week but <2 weeks	16 (10.3)	1 (33.3)	15 (9.9)	
>2 weeks	88 (56.8)	1 (33.3)	87 (57.2)	
Residual stone				
Yes	70 (45.2)	3 (100)	67 (44.1)	0.237
No	53 (34.2)	0 (0)	53 (34.9)	
No CT done	32 (20.6)	0 (0)	32 (21.1)	
Subsequent intervention	59 (38.1)	2 (66.7)	57 (37.5)	0.558

## Discussion

Evaluation of factors associated with urosepsis following PCNL is important as there are modifiable factors that could be avoided and might result in a significant reduction in the incidence and prevalence of urosepsis [9]. Moreover, the aim of the current study was to evaluate factors associated with urosepsis following PCNL.

The mean age of the participants overall was found to be 49.9. More than two-thirds (69.7%) of the participants were males, and the rest were females; all the patients who developed urosepsis were males. The most frequently reported co-morbidity was found to be diabetes mellitus, which was reported in about one-third (34.8%) of the participants and one-third (33%) of the participants with urosepsis found to be diabetic, followed by hypertension, which was reported in 36.8% of the participants and one-third (33%) of those with urosepsis found to also have hypertension, then about 9% were found to have chronic kidney disease, 5.8% were with cardiovascular diseases and others, and this was consistent with the findings reported in the congruent study conducted by Dimitrijevic et al. in which diabetes mellitus was the most common co-morbidity in patients with urosepsis [10].

Regarding the diagnosis and indication of PCNL, the most commonly reported indication was found to be unilateral renal stones, which were reported in more than two-thirds (73.5%) of the participants, and all (100%) of those with urosepsis were found to have unilateral renal stones. Complications of PCNL were reported in only 1.3% of the participants. Previous PCNL was reported in about one-fifth (20%) of the participants. The mean length of stay (in days) was found to be 7.7 days, and this was found to be similar to that reported in the parallel study carried out by Hsiao et al., in which the mean length of stay was found to be nine days [11]. Less than half (41.3%) of the participants had an antibiotic treatment course, and all the participants (100%) with urosepsis had an antibiotic treatment course. The most reported type of stone in the analysis was found to be calcium oxalate in nearly two-thirds (63.2%) of the participants. Slightly less than one-third (29%) received cefuroxime, and one-third (33.3%) of those with urosepsis also received cefuroxime. About 27.1% of the overall participants received ciprofloxacin, and none of those with urosepsis received ciprofloxacin. Bactrim was given to 13 (8.4%) of the participants, and none of the participants with urosepsis received bactrim. The previously mentioned antibiotics were the most commonly reported, as found in the other study conducted by Bischoof et al. [12], in which cefuroxime and ciprofloxacin were used, while other antibiotics used included norfloxacin and nitrofurantoin. The mean duration of antibiotic treatment among overall participants was found to be 8.4 days, and the mean antibiotic treatment among participants with urosepsis was found to be 16.5 days. This was found to be consistent with the findings reported in the study conducted by Klara and Raizada, in which the mean treatment course was found to be 17-22 days [13]. The time of removal of the JJ stent was found to be more than two weeks in more than half (56.8%) of the participants, and 33% of the participants with urosepsis had a JJ stent inserted for more than two weeks, which was found to be consistent with the findings reported in the congruent study carried out by Visser et al., which was carried out three weeks after surgery [14].

The limitations of the study were the fact that the data were collected from one hospital, which restricted the generalizability of the findings. This subject requires more exploration with a bigger sample and the involvement of multiple hospitals in the region to achieve an accurate estimation of the incidence and risk factors for developing urosepsis following percutaneous nephrolithotomy.

## Conclusions

The incidence of urosepsis among the patients who underwent PCNL was less than 2%. Diabetes mellitus, followed by hypertension, were the most prevalent co-morbidities among the participants. Cefuroxime was the antibiotic of choice when treating patients and following urosepsis.
